# On Diphtheritis of the Glans in Some Cases of Paralysis

**Published:** 1852-08

**Authors:** 


*On Diphtheritis of the glans in some cases of paralysis.* By Dr. Hérard.—In two persons, who had been the subjects of cerebral hæmorrhage followed by hemiplegia, Dr. Hérard found a morbid exudation at the extremity of the glans, round the urinary meatus. In both cases, there was incontinence of fæces and urine; and the attention of Dr. Hérard was directed to the glans by the patient complaining of pain at the extremity of the penis. The exudation was removed by careful cleansing, and the use of acid nitrate of mercury.

Dr. Hérard thinks that the origin of the diphtheritic eruption is sufficiently explained, by the penis having been constantly plunged in a metallic vessel containing decomposed urine, and being thus exposed to the emanations therefrom.—*London. Journ. Med.*

